# Dual graph-embedded fusion network for predicting potential microbe-disease associations with sequence learning

**DOI:** 10.3389/fgene.2025.1511521

**Published:** 2025-02-11

**Authors:** Junlong Wu, Liqi Xiao, Liu Fan, Lei Wang, Xianyou Zhu

**Affiliations:** ^1^ College of Computer Science and Technology, Hengyang Normal University, Hengyang, China; ^2^ Technology Innovation Center of Changsha, Changsha University, Changsha, China; ^3^ Hunan Engineering Research Center of Cyberspace Security Technology and Applications, Hengyang Normal University, Hengyang, China

**Keywords:** long and short-term memory networks, graph attention networks, microbe-disease associations, graph convolutional neural networks, full connectivity

## Abstract

Recent studies indicate that microorganisms are crucial for maintaining human health. Dysbiosis, or an imbalance in these microbial communities, is strongly linked to a variety of human diseases. Therefore, understanding the impact of microbes on disease is essential. The DuGEL model leverages the strengths of graph convolutional neural network (GCN) and graph attention network (GAT), ensuring that both local and global relationships within the microbe-disease association network are captured. The integration of the Long Short-Term Memory Network (LSTM) further enhances the model’s ability to understand sequential dependencies in the feature representations. This comprehensive approach allows DuGEL to achieve a high level of accuracy in predicting potential microbe-disease associations, making it a valuable tool for biomedical research and the discovery of new therapeutic targets. By combining advanced graph-based and sequence-based learning techniques, DuGEL addresses the limitations of existing methods and provides a robust framework for the prediction of microbe-disease associations. To evaluate the performance of DuGEL, we conducted comprehensive comparative experiments and case studies based on two databases, HMDAD, and Disbiome to demonstrate that DuGEL can effectively predict potential microbe-disease associations.

## 1 Introduction

Microorganisms play an important and far-reaching role in human life and greatly impact our health (Liang et al., 2018). Recent reports indicate that the human body is host to trillions of microorganisms (Hoffmann et al., 2016) and that the number of microorganisms in the human body far exceeds the number of human cells (Bocci, 1992). These microorganisms constitute the microbiota in the human body (Zhu et al., 2010). The microbiome plays a critical role in human physiology (Heintz-Buschart and Wilmes, 2018), helping the body’s intestinal tract to reduce the growth of pathogenic bacteria and infections and synthesizing some of the vitamins and amino acids needed by the body (Islam et al., 2022). Suppose the microbial community in the human body is out of balance. In that case, it can impair the function of the immune system, increase the risk of infection with pathogens (Pickard et al., 2017), lead to malnutrition or nutritional deficiencies (Burr et al., 2020), and contribute to the development of mental health-related problems such as anxiety and depression (Anisman et al., 2018), as well as metabolic diseases such as obesity and diabetes (Sanz et al., 2015). Of course, the microbiota can help the body regulate and prevent attacks from bacteria outside the body (Barr, 2017); for example, actinomycetes are a class of antibiotic-producing bacteria that produce a wide range of antibiotics such as streptomycin and tetracycline (Grasso et al., 2016). These antibiotics inhibit the growth of other pathogenic microorganisms and help protect the body from infection (Jagannathan et al., 2021). Therefore, predicting potential associations between microorganisms and diseases is vital for unraveling the complex mechanisms of disease occurrence and discovering potential biomarkers (Montaner et al., 2020). By inferring the interactions between microorganisms and diseases, we can better understand the diagnosis and prognosis of diseases and provide new ideas and methods for preventing, diagnosing, and treating diseases (Malla et al., 2019). As technology advances, we no longer rely solely on traditional biological methods to explore the association between microbes and disease (Gilbert et al., 2016). Instead, we are increasingly introducing computational modelling into our research to predict the role of microbes in disease occurrence, development, and treatment through techniques such as big data analytics and deep learning (Marcos-Zambrano et al., 2021), which are more practical and accurate (Najafabadi et al., 2015). Researchers have recently established a series of microbe-disease association databases to conduct an in-depth study of the potential link between microbes and diseases (Jin et al., 2022). These databases combine a large amount of microbial composition data and disease information. For example, the HMDAD database created by Ma et al. became the first to document human microbe-disease associations by manually organizing a large amount of public literature (Ma et al., 2017). This database covers 483 pieces of information about the association between 39 diseases and 292 microorganisms. Second, Janssens et al. created a microbial-disease association database called Disbiome by collecting 10,922 experimental records from 1,191 documents containing 372 diseases and 1,622 microorganisms (Janssens et al., 2018).

Researchers can explore and discover the relationships between microorganisms and different diseases using the above microbe-disease association database as the primary data. Moreover, these recent technological tools can be broadly categorized into four types, namely, network-based methods (Wu et al., 2018), matrix decomposition-based methods (Shen et al., 2021), traditional machine learning-based methods (Afshari et al., 2022), and graph neural network-based methods (Li et al., 2023).

In DuGEL, we use both Graph Convolutional Neural Network (GCN) and Graph Attention Network (GAT), where GCN is specifically designed to process graph data (Jin et al., 2021). GCN can learn feature representations at both node and graph levels, and it achieves the task of learning and predicting the representations of graph data by efficiently exploiting the connectivity relationships between the nodes (Zhou et al., 2023). By adaptively learning the attention weights between each node and its neighbouring nodes, GAT can better capture local structural information in graph data. The GAT introduction enriches the representational capabilities of the graph neural network, allowing it to perform well when dealing with complex graph-structured data (Munikoti et al., 2023). DuGEL can adapt to an extensive range of datasets with solid robustness.

Unlike the above methods, in this paper, we designed a new computational model called DuGEL based on the graph convolutional neural network and the graph attention network to infer possible microbe-disease associations. In DuGEL, we first downloaded known microbe-disease associations to form a heterogeneous microbe-disease network. Then, we input this network into a graph convolutional neural network and a graph attention network separately to obtain the local and global features of nodes in the network. Next, we spliced the outputs of the graph convolutional neural network and the graph attention network and then introduced a Long Short-Term Memory (LSTM) network to process the fused features. Finally, the output of the LSTM would be passed to a fully connected layer to infer potential associations between microbes and diseases. Experiments showed that DuGEL obtained satisfactory predictive performance with a 5-fold cross-validated auc of 0.9698 and 0.9119 for HMDAD and Disbiome datasets, respectively, and may be a potential tool for future microbe-disease association prediction.

## 2 Materials and methods

### 2.1 Datasets

HMDAD, constructed by Ma et al. (2017), and Disbiome (Janssens et al., 2018), constructed by Janssens et al., are the main publicly available biomedical databases containing microbe-disease association data. As shown in Table 1, HMDAD database covers 483 known microbe-disease associations, and processing these data, we ended up with 450 known microbial-disease associations. The HMDAD database provides a valuable information resource for studying microbial-disease relationships. In addition, the Disbiome, constructed by Janssens et al., is a publicly available database of microbe-disease associations. As shown in Table 1, Disbiome database collects 5,573 known associations from published academic papers for 240 diseases and 1,098 microorganisms. After de-duplication, we had 4,351 known microbe-disease associations covering 218 diseases and 1,052 microorganisms. Due to its extensive data collection and detailed information records, the Disbiome database has become a vital data support for research in this field.

**TABLE 1:** The statistics of the two databases.

Datasets	Microbes	Diseases	Associations
HMDAD	292	39	450
Disbiome	1,052	218	4,351

After acquiring the initial data, we performed data preprocessing steps to ensure the quality of the data and the validity of the model training. First, we removed all duplicate records to ensure that the association of each microbe with a disease was unique. Further, we converted the data into a uniform format to facilitate subsequent processing and model training. For simplicity, for each dataset, let $M=\left\{m_{1}m_{2},\ldots ,m_{N_{M}}\right\}$ denote the set of newly downloaded different microorganisms, and $D=\left\{d_{1}d_{2},\ldots ,d_{N_{d}}\right\}$ represent the set of newly downloaded different diseases. Thus, we can construct a primitive known microbe-disease association network $Net=\langle M\cup \;D,E\rangle$ as follows: for any given $m_{i}\left(1\leq i\leq N_{m}\right)$ and $d_{j}\left(1\leq j\leq N_{d}\right)$ , if and only if there is a known association between them, we assume that there is an edge belonging to E . Obviously, based on above definition, we can obtain an adjacency matrix $A\in R^{N_{m}\times N_{d}}$ as follows: for any given $m_{i}\left(1\leq i\leq N_{m}\right)$ and $d_{j}\left(1\leq j\leq N_{d}\right)$ if and only if there is an edge between them in E , there is $A_{i,j}=1$ , otherwise, there is $A_{i,j}=0$ .

### 2.2 Multiple similarity calculation of disease

#### 2.2.1 Gaussian interaction profile kernel similarity of disease

Based on the assumption that two similar diseases will show similar interaction and non-interaction relationships with the same microorganism, in this section, we adapt the Gaussian interaction profile kernel similarity between a pair of diseases $d_{i}$ and $d_{j}$ as follows:

$$GD\left(d_{i},d_{j}\right)=\exp\left(-\lambda_{d}{\left\|A\left(i,:\right)-A\left(j,:\right)\right\|}^{2}\right)$$

Where $A\left(i,:\right)$ and $A\left(j,:\right)$ represent the $i^{th}$ and $j^{th}$ rows of the adjacency matrix A respectively, and $\lambda_{d}$ denotes the normalized kernel bandwidths that can be calculated as follows:

$$\lambda_{d}=\frac{1}{\left(\frac{1}{n_{d}}\sum_{i=1}^{n_{d}}\left\|A\right.\left(i,:\right){\|}^{2}\right)}$$

#### 2.2.2 Cosine similarity of disease

Based on the assumption that if two diseases are similar to each other, then their cosine curves will be more coincident, we introduce the cosine similarity between a pair of diseases $d_{i}$ and $d_{j}$ as follows:

$$CD\left(d_{i},d_{j}\right)=\frac{A\left(i,:\right)\cdot A\left(j,:\right)}{\left|A\left(i,:\right)\right|\times \left|A\left(j,:\right)\right|}$$

The result of cosine similarity has good stability and certainty, the calculation speed is fast and the result is more intuitive. Suitable for large-scale information retrieval. Where $A\left(i,:\right)\cdot A\left(j,:\right)$ denotes multiplying the vectors of row i and row j , $\left|A\left(i,:\right)\right|$ represents the mode of $A\left(i,:\right)$ , and $\left|A\left(j,:\right)\right|$ represents the mode of $A\left(j,:\right)$ . $\left|A\left(i,:\right)\right|*\;\left|A\left(j,:\right)\right|$ represents the multiplication of two moduli, and then the vector’s product removes the modulus’s value. Finally, the cosine value of the angle between the two diseases is obtained, that is the cosine similarity. The calculation result of cosine similarity is between −1 and 1. When the similarity between two diseases is exceptionally high, the calculation result tends to be 1. When the similarity between two diseases is very low, the calculation result tends to −1.

#### 2.2.3 Functional similarity of disease

Based on the assumption that similar diseases tend to interact with similar genes, in this section, we calculate the disease functional similarity based on the functional associations between disease-related genes as follows: Firstly, we download the gene interactions from the HumanNet database in which, every interaction has an associated log-likelihood score (LLS). And then, for any given diseases $d_{i}$ and $d_{j}$ , let $G_{i}=\left\{g_{i_{1}},g_{i_{2}},\ldots ,g_{i_{m}}\right\}$ and $G_{j}=\left\{g_{j_{1}},g_{j_{2}},\ldots ,g_{j_{n}}\right\}$ be the gene sets of $d_{i}$ and $d_{j}$ separately, we will define the functional similarity between $d_{i}$ and $d_{j}$ as follows:

$$DFS\left(d_{i},d_{j}\right)=\frac{\sum_{g_{k}\in G_{i}}F_{G_{j}}\left(g_{k}\right)+\sum_{g_{k}\in G_{j}}F_{G_{i}}\left(g_{k}\right)}{m+n}$$

where $F_{G_{t}}\left(g_{q}\right)=\max_{g_{p}\in G_{t}}\left(FSS\left(g_{p},g_{q}\right)\right)$ and $FSS\left(g_{p},g_{q}\right)$ is the functional similarity score between the genes $g_{p}$ and $g_{q}$ , which can be calculated as follows:

$$\text{FSS}\left(g_{p},g_{q}\right)=\left\{\begin{array}{cc} 1 & \text{if }p=q\\ \frac{LLS\left(g_{p},g_{q}\right)-LLS_{\min}}{LLS_{\max}-LLS_{\min}} & \text{if }p\neq q \end{array}\right.$$

where ${\text{LLS}}_{\max}$ and ${\text{LLS}}_{\min}$ represent the maximum value of LLS and the minimum value of LLS in HumanNet, respectively.

Thereafter, by combining the above GIP kernel similarity, disease cosine similarity, and functional similarity of disease, we can obtain an integrated similarity matrix of disease as follows:

$$DS=\frac{GD+CD+DFS}{3}$$

### 2.3 Multiple similarity calculation of microbe

#### 2.3.1 Gaussian interaction profile kernel similarity of microbe

In the same way, we can calculate the gaussian interaction profile kernel similarity between any two microbes $m_{i}$ and $m_{j}$ as follows:

$$MD\left(m_{i},m_{j}\right)=\exp\left(-\lambda_{m}{\left\|A\left(:,i\right)-A\left(:,j\right)\right\|}^{2}\right)$$

where $A\left(:,i\right)$ and $A\left(:,j\right)$ represent the $i^{th}$ and $j^{th}$ columns of the adjacency matrix A respectively, and $\lambda_{m}$ denotes the normalized kernel bandwidths that can be calculated as follows:

$$\lambda_{m}=\frac{1}{\left(\frac{1}{n_{m}}\sum_{i=1}^{n_{m}}\left\|A\right.\left(:,i\right){\|}^{2}\right)}$$

#### 2.3.2 Cosine similarity of microbe

Similarly, the cosine similarity between any two microbes $m_{i}$ and $m_{j}$ can be obtained as follows:

$$CM\left(m_{i},m_{j}\right)=\frac{A\left(:,i\right)\cdot A\left(:,j\right)}{\left|A\left(:,i\right)\right|\times \left|A\left(:,j\right)\right|}$$

The calculation process of cosine similarity between two microorganisms is the same as that of disease cosine similarity. Similarly, when the similarity between two microorganisms is exceptionally high, the calculation result tends to be 1. When the similarity between two microorganisms is very low, the calculation result tends to −1.

#### 2.3.3 Functional similarity of microbe

The functional similarity of the microbe is calculated by using the following method (Zhang et al., 2018): for any given disease $d^{t}$ , it is first raised Directed Acyclic Graph $DAG_{d_{t}}=\left(V_{d_{t}},E_{d_{t}}\right)$ , where $V_{d_{t}}$ includes the disease $d_{t}$ and its ancestor diseases, $E_{d_{t}}$ contains all the directed edges from parent nodes to children nodes, and then, the semantic contribution of the disease $d_{l}$ in $V_{d_{t}}$ to $d_{t}$ is defined as:

$$SC_{d_{t}}\left(d_{l}\right)=\left\{\begin{array}{cc} 1 & \text{ if }d_{l}=d_{t}\\ \max\left\{0.5\times SC_{d_{l}}\left(d_{l}^{\prime }\right)|d_{l}^{\prime }\in \text{children of }d_{l}\right\} & \text{ otherwise } \end{array}\right.$$

The semantic value of disease $d_{t}$ is formulated by:

$$SV_{d_{t}}=\sum_{d_{l}\in V_{d_{t}}}SC_{d_{t}}\left(d_{l}\right)$$

Then, the semantic similarity between any two diseases $d_{i}$ and $d_{j}$ can be defined as follows:

$$DSS\left(d_{i},d_{j}\right)=\frac{\sum_{d_{i}\in V_{d_{i}}\cap V_{d_{j}}}\left(SC_{d_{i}}\left(d_{l}\right)+SC_{d_{j}}\left(d_{l}\right)\right)}{SV_{d_{i}}+SV_{d_{j}}}$$

Relying on the above formulae, we can further define the similarity between the disease $d_{i}$ and a set of diseases D as follows:

$$\text{DS}\left(d_{i},D\right)=\max_{d_{j}\in D}\left(\text{DSS}\left(d_{i},d_{j}\right)\right)$$

Hence, for any two given microbes $m_{i}$ and $m_{j}$ , we can calculate the function similarity between them as follows:

$$MFS\left(m_{i},m_{j}\right)=\frac{\sum_{d_{j}\in D_{j}}DS\left(d_{j},D_{i}\right)+\sum_{d_{j}\in D_{i}}DS\left(d_{j},D_{j}\right)}{\left|D_{i}\right|+\left|D_{j}\right|}$$

where $D_{i}$ denotes the set of diseases associated with the microbe $m_{i}$ , and $D_{j}$ represents the set of diseases associated with the microbe $m_{j}$ .Obviously, by combining the above GIP kernel similarity, disease cosine similarity, and functional similarity of the microbe, we can obtain an integrated similarity matrix of the microbe as follows:

$$MS=\frac{MD+CM+MFS}{3}$$

### 2.4 Construction of the heterogeneous network

Based on above descriptions, it is easy to see that we can construct a heterogeneous network Y by combining the integrated similarity matrix DS of disease and the integrated similarity matrix MS of microbe with the adjacency matrix A as follows:

$$Y=\left[\begin{array}{c} DS\\ A^{T} \end{array}\begin{array}{c} A\\ MS \end{array}\right]$$

### 2.5 Structure of the DuGEL

**FIGURE 1:**
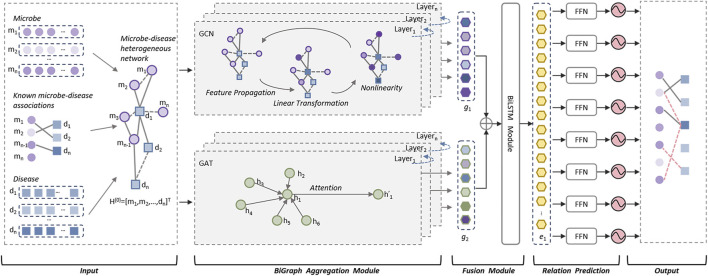
The structure of DuGEL.

As illustrated in above Figure 1, the DuGEL consists of the following five steps:

• Step 1: Construct a heterogeneous microbe-disease network based on newly downloaded known microbe-disease associations and multiple microbe and disease similarity metrics.
• Step 2: Feeding the heterogeneous microbe-disease network forward into a dual channel structure consisting of a Graph Convolutional Neural Network (GCN) and a Graph Attention Network (GAT), where the GCN is utilized to extract spatial features of nodes in the heterogeneous microbe-disease network from local to global, and the GAT is adopted to assign different importance to the neighbors of each node in the heterogeneous microbe-disease network as it is processed.
• Step 3: Splicing the outputs of GCN and GAT by simply fusing the information captured by GCN and GAT and combining structural and node characteristics and the importance between neighboring nodes.
• Step 4: Implementing a Long Short-Term Memory (LSTM) network to process the fused features, then feeding the output of LSTM into a fully connected layer to convert the high-level features captured by LSTM into the target output space.
• Step 5: By feeding the newly obtained feature vectors of the target output space into a Sigmoid function for binary prediction, potential associations between microbes and diseases can be finally computed.

### 2.6 Microbe-disease representation layer

In DuGEL, the input layer is the Microbe-Disease Association Representation Layer, is a component used to convert raw data of known microbe-disease associations into a structured data fame that can be processed by subsequent graph neural networks. Firstly, the newly collected microbe-disease association data need to be pre-processed to ensure consistency and accuracy. The preprocessed data will be used in turn to construct a binary microbe-disease association matrix *M*, which implies potential relationships between microorganisms and disease (Marsh and Zaura, 2017), and can be defined as follows: Given a microorganism m and a disease n , he known relationship between the microorganism and the disease can be characterized by a binary association matrix $M\in R^{m\times n}$ , where the matrix element $M_{ij}$ is 1 if there is a known association between the microorganism i and the disease j , and 0 otherwise. Each row of the matrix represents a microorganism, and each column represents a disease. The entries in the matrix indicate the presence or absence of an association. The graph structure G is obtained by the association matrix M , where the nodes in the graph are either microbe nodes or disease nodes, and if there is an association between a microbe node and a disease node (Abuin-Denis et al., 2024), i.e., $M_{ij}=1$ , then an edge exists between the nodes.

To enhance the prediction ability of DuGEL, similarity information is also fused in the representation layer (Feng et al., 2023), which contains the microbial similarity matrix $S_{m}$ and the disease similarity matrix $S_{d}$ . Among them, the microbial similarity matrix $S_{m}$ captures similarities between different microorganisms based on genomic, phenotypic, or ecological characteristics. After the microbial similarity matrix $S_{m}$ and disease similarity matrix $S_{d}$ are constructed, we fuse this similarity information with the microbe-disease association matrix M to enhance the performance of the association prediction model. Therefore, at this point, we can obtain the association matrix that incorporates microbial similarity and disease similarity as a graph structure (Yu et al., 2024), which further serves as the initial input form for the dual graph-based feature extraction module. This graph structure captures direct associations and enables the model to learn a more comprehensive representation of features.

In addition, we will initialize each representative microbe or disease node in the graph with a feature vector. Formally, let $X_{m}\in R^{m\times d}$ represent the initial feature matrix for microbes and $X_{d}\in R^{n\times d}$ represent the initial feature matrix for diseases, where d is the dimension of the feature vector. The initial embeddings $X_{m}$ and $X_{d}$ are combined into a unified feature matrix $X\in R^{\left(m+n\right)\times d}$ . X is fed forward as input to the dual graph-based feature extraction module.

The microbe-disease association representation layer lays the foundation for the entire DuGEL model, and by meticulously structuring the input data, the layer ensures that subsequent graph neural networks are able to effectively capture both local and global patterns in the data. The construction of the graph enables the model to fully utilize all available information, thereby improving the accuracy and robustness of microbe-disease association predictions.

### 2.7 Bipartite graph feature extraction module

In this study, the dual graph feature extraction module is a core component of the DuGEL designed to extract deep features from the input microbe-disease association feature matrix. The module combines two parallel graph neural network architectures: graph convolutional network (GCN) and graph attention network (GAT), ensuring effective capture of local and global relationships in the graph.

#### 2.7.1 Graph convolution sublayer

The Graph Convolutional Network (GCN) module extracts the spatial features of the graph by processing the microbe-disease feature matrix X to capture the intrinsic structural information of the graph. The spatial features represent the connections between nodes, including direct links (edges) and indirect influences through neighboring nodes. For example, in a microbe-disease graph, the direct association between a microbe and a disease, as well as second- or higher-order neighborhood relationships, contribute to the spatial information. The GCN extracts the features of the nodes by applying convolutional operations on the graph structure, and aggregates the information of the aggregated features of the local neighborhoods of each node by means of the layer-wise propagation rules (Du et al., 2024). The propagation rules of the GCN layers are defined as follows:

$$H^{\left(l+1\right)}=\sigma \left({\hat{D}}^{-\frac{1}{2}}\hat{A}{\hat{D}}^{-\frac{1}{2}}H^{\left(l\right)}W^{\left(l\right)}\right)$$

where $H^{\left(l\right)}$ is the node representation X of the l nd layer, $W^{\left(l\right)}$ whose initial input is, is the weight matrix of the l -th layer, σ denotes the nonlinear activation function, $\hat{A}$ is the representation of the adjacency matrix A plus the unitary matrix I , and $\hat{D}$ is the corresponding $\hat{A}$ degree matrix.

GCN effectively smoothies the feature representation over the graph structure and ensures that the representation of each node is influenced by its neighbors (Chen et al., 2021), thus capturing the local structure information. By aggregating a node’s neighbor information, the feature representation of a node is made to reflect its local graph structure. This aggregation operation is performed in each layer, and through the gradual aggregation of multiple layers (Liu et al., 2018), the GCN can capture a wider range of graph information. This is particularly important for microbe-disease association prediction, as some associations may not be directly visible, but indirectly inferred through multi-hop relationships.

#### 2.7.2 Attention sublayer

The Graph Attention Network (GAT) module introduces an attention mechanism that assigns different importance coefficients to each node’s neighbors. For each node i in the graph, GAT computes an attention coefficient $\alpha_{i,j}$ between node i and its neighbor node j , which is learned by a shared attention mechanism:

$${\;\alpha }_{ij}=\frac{\exp\left(LeakyReLU\left(a^{T}\left[W^{\left(l\right)}h_{i}∥W^{\left(l\right)}h_{j}\right]\right)\right)}{\sum_{k\in N\left(i\right)}\exp\left(LeakyReLU\left(a^{T}\left[W^{\left(l\right)}h_{i}∥W^{\left(l\right)}h_{k}\right]\right)\right)}$$

where $h_{i}$ and $h_{j}$ are the feature vectors of node i and node j , respectively; $W^{\left(l\right)}$ is the learnable weight matrix; ∥ represents the splicing operation between the vectors, $N\left(i\right)$ denotes the set of neighboring nodes of node i , and $a^{T}$ is the weight vector of the attention mechanism. Further, the updated feature vector $h_{i}^{\prime }$ of node i is computed by weighted sum of its neighboring features:

$$h_{i}^{\prime }=\sigma \left(\sum_{j\in N\left(i\right)}\alpha_{ij}W^{\left(l\right)}h_{j}\right)$$

The GAT sublayer enables the model to focus on the most relevant parts of the graph, thus capturing the importance of each neighboring node during the feature aggregation process. The advantage is that it can dynamically adjust the contribution of each neighbor to a node’s feature update, and by learning different attention weights, GAT can allocate different attention between different parts of the graph structure (Chatzianastasis et al., 2023). This is particularly useful when dealing with complex biological networks, where associations between microorganisms and diseases may have different biological significance and importance.

The dual graph feature extraction module can capture local and global information in the graph structure by combining GCN and GAT approaches. GCN emphasizes the aggregated features of a node’s local neighbors. At the same time, GAT dynamically adjusts the weights of the neighboring nodes through the attentional mechanism (Wang et al., 2022), thus providing more flexible and fine-grained control in the feature extraction process. This dual approach ensures that the model understands direct associations between microbes and diseases and identifies potential indirect relationships through graph structural features and attention weights. Combining these two approaches enables the DuGEL model to excel in microbe-disease association prediction tasks, providing more accurate and comprehensive predictions.

The dual graph feature extraction module plays a crucial role in the DuGEL model. Capturing complex graph structure information improves the model’s predictive ability and enhances its robustness and generalization ability.

### 2.8 Feature fusion and sequence learning networks

#### 2.8.1 Fusion layer

After processing in the GCN and GAT layers, we obtain two sets of feature representations $H_{GCN}\in R^{\left(m+n\right)\times d}$ and $H_{GAT}\in R^{\left(m+n\right)\times d}$ . These two sets of feature representations capture the spatial properties of the graph and the weighted properties of the important nodes, respectively. For integrating the feature representations generated by the GCN and the GAT, we introduce the fusion layer. These representations encode complementary information: GCN captures spatial structure, while GAT focuses on the relative importance of neighboring nodes.

In the dual graph feature extraction module, we extract two different feature representations through GCN and GAT. The task of the feature fusion layer is to effectively fuse these different features to obtain a comprehensive feature representation. The fusion operation can be realized in various ways, such as concatenation, weighted summing, or multilayer perceptron (MLP) (Afzal et al., 2023). Here, we adopt the concatenation operation to stitch together feature representations from different sub-networks to form a richer feature vector. Assuming that the feature representation extracted through GCN is $H_{GCN}$ and the feature representation extracted through GAT is $H_{GAT}$ , the fused feature representation $H_{\text{fusion}\;}$ :

$$H_{\text{fusion}\;}=\left[H_{GCN}\left|\right|H_{GAT}\right]$$

where ∥ represents the concatenation operation between the vectors; the fused feature matrix $H_{\text{fusion}\;}{\in R}^{\left(m+n\right)\times 2d}$ contains features extracted from two different viewpoints, providing a more comprehensive node representation.

#### 2.8.2 Sequence learning layer

**FIGURE 2:**
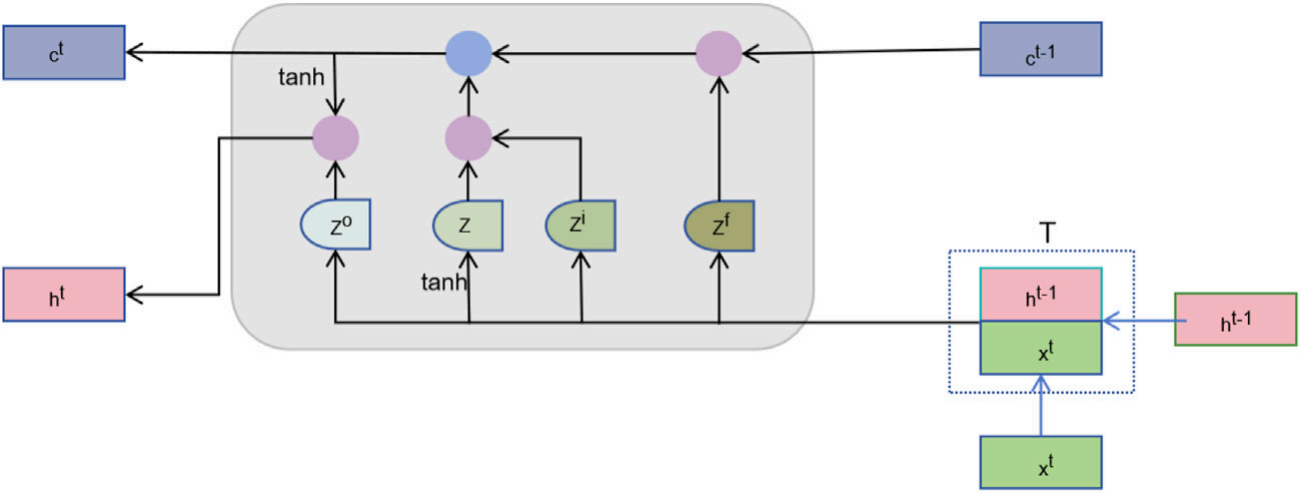
The structure of LSTM.

After feature fusion, we need to further learn useful information from these high-dimensional features. The sequence learning layer is designed to capture the temporal or sequential dependencies between features to enhance the prediction capability (Yuan et al., 2023). By treating the node features as a sequence, the order in which node features are fed to the LSTM introduces a dependency chain. The model learns how the features of one node are influenced by or related to those of neighboring nodes. As illustrated in Figure 2, we introduce the Long Short-Term Memory Network (LSTM), which is capable of effectively remembering long-term dependencies and is suitable for processing sequence data (Yoo et al., 2023). The LSTM processes each node’s features across multiple time steps. Here, the “time steps” correspond to sequential relationships between node features derived from their embedding in the heterogeneous network. We represent the vector corresponding to the t -th time step in the matrix $H_{\text{fusion}\;}{\in R}^{\left(m+n\right)\times 2d}$ as $H_{fusion}^{t}{\in R}^{2d}$ . The computational mechanism of the LSTM can be formally represented as follows:

$$I_{t}=\sigma \left(W_{i}H_{fusion}^{t}+U_{i}h_{t-1}+b_{i}\right)$$

$${\;f}_{t}=\sigma \left(W_{f}H_{fusion}^{t}+U_{f}h_{t-1}+b_{f}\right)$$

$$o_{t}=\sigma \left(W_{o}H_{fusion}^{t}+U_{o}h_{t-1}+b_{o}\right)$$

$${\tilde{c}}_{t}=\tanh\left(W_{c}H_{fusion}^{t}+U_{c}h_{t-1}+b_{c}\right)$$

$$C_{t}=f_{t}⊙c_{t-1}+i_{t}⊙{\tilde{c}}_{t}$$

$$h_{t}=o_{t}⊙\tanh\left(c_{t}\right)$$

where $i_{t}$ , $f_{t}$ , and $o_{t}$ denote the activation vectors of the input, oblivion, and output gates, respectively, ${\tilde{c}}_{t}$ is the candidate cell state, $c_{t}$ and $h_{t}{\in R}^{d_{l}}$ are the cell state and hidden state at time step, respectively, W and U are the learnable weight matrices, and b is the bias vector. $d_{l}$ is the dimensionality of the LSTM hidden layer. The output of the LSTM $H_{\text{LSTM}\;}={\left\{d_{l}^{t}\right\}}_{t=1}^{m+n}{\in R}^{\left(m+n\right)\times d_{l}\;}$ combines information provided by the feature fusion layer, and captures through sequence learning the complex dependencies between features.

The feature fusion layer effectively integrates feature representations extracted from different perspectives, providing more comprehensive input data (Zhang et al., 2020). The sequence learning layer, on the other hand, further enhances the model’s predictive capability by capturing the temporal dependencies between features. Combining the two ensures the model can fully utilize all available information to achieve higher accuracy and robustness in microbe-disease association prediction tasks.

### 2.9 Prediction

The feature representation $H_{\text{LSTM}\;}$ output from the sequence learning layer is used as input to the fully connected layer for further processing. The fully connected layer maps the high-dimensional features to the final prediction results through a series of linear transformations and nonlinear activation functions (Feng et al., 2023). The computational process of the fully connected layer can be formally represented as:

$$\hat{y}=\sigma \left(W_{fc}H_{LSTM}+b_{fc}\right)$$

where $W_{fc}$ and $b_{fc}$ are the weights and bias parameters of the fully connected layer, respectively, and σ represents the Sigmoid nonlinear activation function.

Ultimately, the output layer gives the predicted probability of microbe-disease association (Marsh and Zaura, 2017). By setting an appropriate threshold, it is possible to determine whether there is an association between microbes and diseases.

### 2.10 Objective function

To measure the difference between the predicted and true values of the model, in the DuGEL model, we use the cross-entropy loss function to evaluate the effectiveness of microbe-disease association prediction (Mao et al., 2023). The cross-entropy loss function is commonly used in classification problems, and in microbe-disease association prediction, we modeled the problem as a binary classification task, i.e., predicting whether there is an association between a certain pair of microorganisms and a disease. The cross-entropy loss function is defined as follows:

$$L_{CE}=-\frac{1}{N}\sum_{i=1}^{N}\left[y_{i}\log\left({\hat{y}}_{i}\right)+\left(1-y_{i}\right)\log\left(1-{\hat{y}}_{i}\right)\right]$$

where N denotes the number of samples; $y_{i}$ is the true label of the i -th sample, indicating the presence of an association, and 0 denotes the absence of an association. ${\hat{y}}_{i}$ is the predicted probability of the model for the i -th sample, indicating the probability of an association between microorganisms and diseases. The cross-entropy loss function improves the accuracy of the prediction by penalizing the wrong prediction of the model, which makes the model continuously adjust the parameters during the training process.

To prevent model overfitting, we add a regularization term to the loss function. The regularization term improves the generalization ability of the model by adding a penalty to the model complexity in the loss function (Fu et al., 2023), encouraging the model to choose simpler parameter configurations. In the DuGEL model, we use L2 regularization or weight decay. It is defined as follows:

$$L_{\text{reg}\;}=\sum_{k}{∥W_{k}∥}_{2}^{2}$$

where $W_{k}$ denotes the k rd weight matrix of the model; and ${∥W_{k}∥}_{2}^{2}$ is the L2 paradigm of $W_{k}$ , i.e., the sum of squares of the weight matrix. The regularization term prevents the model from overfitting the training data by penalizing excessively large values of the weights, thus improving the model’s performance on the test data.

Ultimately, the integrated loss function of the DuGEL model consists of a cross-entropy loss and a regularization term of the following form:

$$L=L_{\text{CE}}+{\lambda \;*\;L}_{\text{reg}\;}$$

where λ is the number, which controls the weights of the regularization term. This integrated loss function takes into account both the accuracy of the model prediction and the complexity of the model, and by balancing the two (Cui et al., 2019), it ensures that the model not only accurately fits the training data during training, but also has good generalization ability.

## 3 Experiments and results

In this section, we will detail the experimental setup, evaluation metrics, and baseline methodology used to evaluate the performance of the DuGEL model and present the experimental results and analysis. The effectiveness and superiority of the DuGEL model in the microbe-disease association prediction task are verified by comparing it with multiple baseline methods. The corresponding pseudo-code of the DuGEL model is shown in Table 2.

**TABLE 2:** Pseudocode of the DuGEL model proposed in this study.

Algorithm 1 Algorithm of the DuGEL.
Input: Known associations matrix $A\in R^{N_{m}\times N_{d}}$ ;
$\hat{A}$ : Representation of the adjacency matrix A plus the unitary matrix I
Microbe similarity matrix $S_{m}\in R^{N_{m}\times N_{m}}$
Disease similarity matrix $S_{d}\in R^{N_{d}\times N_{d}}$
N : the number of iterations for DuGEL
L : the number of layers in GCN and GAT
Output: Reconstructed microbe-disease associations matrix $A^{\prime }$
1 Phase 1: Construct the heterogeneous network $Y\in R^{\left(N_{m}+N_{d}\right)\times \left(N_{d}\times N_{m}\right)N_{d}\times N_{d}}$
2 $Y=\left[S_{m},A,A^{T},S_{d}\right]$
3 Phase 2: Initialize the embedding feature matrix $H_{0}$
4 $H_{0}$ = Initial embeddings for microbes and diseases
5 Phase 3: for i = 1 to N do
6 for l = 1 to L do
7 Calculate the embedding feature $H_{l}\left(l=1,...,L\right)$ in the *l*th layer according to the GCN formula and GAT:
8 $H_{l}^{GCN}=\sigma \left(D^{-0.5}*\;\hat{A}*\;D^{-0.5}*\;H_{l-1}*\;W_{l}\right)$
9 Update $H_{l}$ using GAT mechanism:
10 $H_{l}^{GAT}$ = Attention ( $H_{l}$ )
11 end for
12 end for
13 Phase 4: Feature Fusion and Sequential Learning
14 Concatenate the final embeddings of microbes and diseases:
15 $H_{fused}=\text{Concatenate}\left(H_{l}^{GCN},H_{l}^{GAT}\right)$
16 Pass $H_{fused}$ through a BiLSTM layer:
17 $H_{seq}$ = BiLSTM( $H_{fused}$ )
18 Apply a dense layer to the output of BiLSTM:
19 $H_{final}$ = Dense ( $H_{seq}$ )
20 Phase 5: Reconstruct Associations
21 Compute the reconstructed association matrix $A^{\prime }$ using the final embeddings:
22 $A^{\prime }=\text{sigmoid}\left(H_{final}\right)$
23 Return $A^{\prime }$

### 3.1 Experimental setup

**FIGURE 3:**
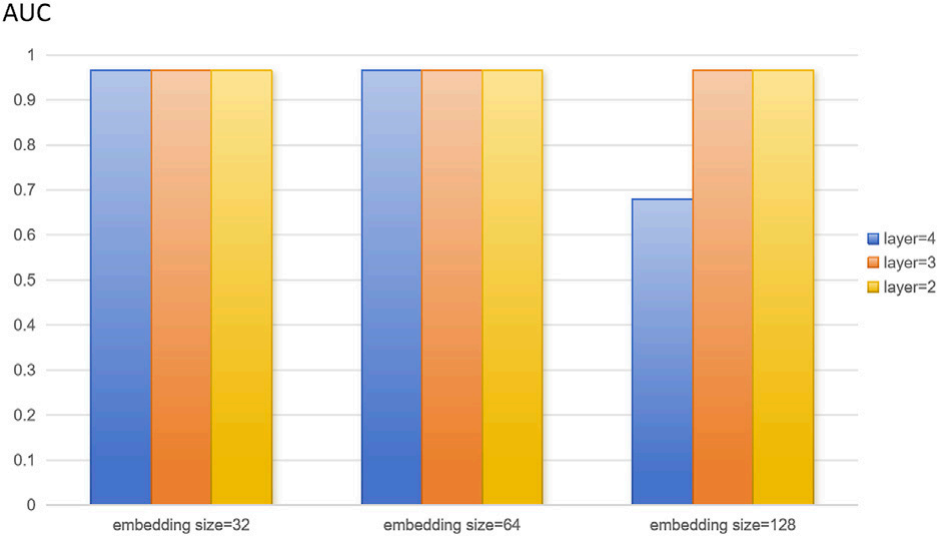
Model parameters analysis on the HMDAD dataset.

**FIGURE 4:**
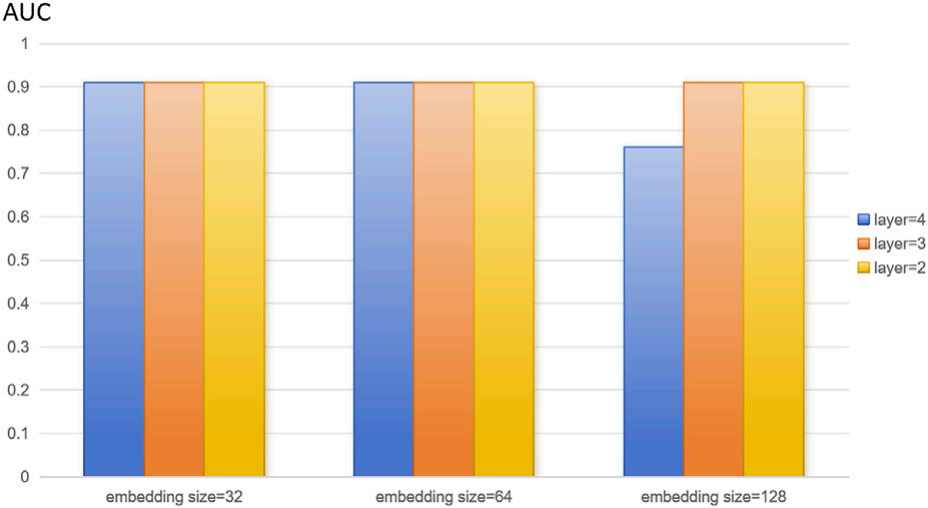
Model parameters analysis on the disbiome dataset.

In this study, we extracted disease features, microbial features, and disease-microbe association matrices from the HMDAD and Disbiome databases, and the three feature matrices mentioned above were used to construct heterogeneous maps to reflect the interactions between diseases and microbes for disease characterization and microbial characterization. The number of training rounds was set to 4,000 in the training phase. To optimize the algorithm to adjust the weights, the learning rate was set to 0.01. We set the random deactivation strategy for the adjacency matrix with the dropout ratio set to 0.5, thus preventing overfitting. For the subject model, to randomly discard some network connections during the training process to enhance the model’s generalization ability, we similarly set the random deactivation strategy with the dropout ratio set to 0.5. In addition, we set the similarity weight to 6 to weigh the similarity features of diseases and microorganisms. From the above description, it is easy to see that there are several hyperparameters in DuGEL, such as the dimension k of the embedded features, the number of layers L, the initial learning rate r of the optimizer, the total training epoch α, the node dropout β, and the rule dropout γ. As illustrated in Figures 3, 4, the various results for several combinations of parameters k and L in the 5-fold cv. From the figures, it is easy to see that the optimal combination of k and L is L = 2, k = 128, which indicates that the first and second-layer embedded features contain more information than the third-layer embedded features. After analyzing, this may be due to excessive smoothing of LSTM.

In the training and evaluation phases of the model, we perform multiple cross-validations. In each validation, the dataset is divided into a training set and a test set, which trains the model on the training set and evaluates the model performance on the test set. Specifically, 5-fold cross-validation (k_folds) is used to evaluate the model performance. In each cross-validation, the data are randomly disrupted and divided into five parts, one used as the test set and the rest as the training set. To ensure the stability and reliability of the results, we repeat the execution of the experiment 5 times and report the average performance metrics.

### 3.2 Evaluation metrics

In order to evaluate the method proposed in this paper, we employ a series of evaluation metrics to comprehensively measure the performance of the model, including AUC, accuracy and specificity The formal definitions and calculations of each evaluation metric are given below:

Accuracy is the ratio of the number of correctly categorized samples to the total number of samples, which is defined as follows:

$$\text{Accuracy}=\frac{TP+TN}{TP+TN+FP+FN}$$

where TP denotes True Positives, FP denotes False Positives, FN denotes False Negatives, and TN denotes True Negatives. Accuracy reflects the overall ability of the model to correctly classify.

AUC (Area Under the ROC Curve) represents the area under the receiver operating characteristic curve (ROC Curve), which is used to measure the classification performance of the model. The ROC Curve plots the True Positive Rate (TPR) and False Positive Rate (FPR) through different thresholds. TPR and FPR are defined as follows:

$$\text{TPR}\left(\text{Recall}\right)=\frac{TP}{TP+FN}$$

$$\text{FPR}=\frac{FP}{FP+TN}$$

The value of AUC is between 0 and 1, with larger values indicating better model performance.

Specificity, also known as True Negative Rate (TNR), is formally defined as follows:

$$\text{Specificity}=\frac{TN}{FP+TN}$$

Specificity indicates the proportion of all samples that are actually negative that are correctly predicted to be negative.

### 3.3 Alternative methods for microbedisease association prediction

In order to compare the methods proposed in this paper horizontally, as shown in Table 3, we introduced nine microbe-disease association prediction methods of.

**TABLE 3:** Microbe-disease association prediction methods.

Method	Approach
KATZHMDA (Chen et al., 2017)	Use of KATZ measurements to infer possible microbe-disease associations
LRLSHMDA (Wang et al., 2017)	Microbial disease prediction using the Laplace regularised least squares framework
NTSHMDA (Luo and Long, 2018)	Predicting potential microbe-disease associations using a model based on stochastic roaming restarts
BiRWMP (Shen et al., 2018)	Predicting microbial-disease using a bidirectional stochastic wandering approach
NBLPIHMDA (Wang et al., 2019)	Detecting potential microbe-disease associations using a bidirectional tag dissemination scheme
HMDApred (Fan et al., 2020)	Predicting microbe-disease associations using network-consistent projections and multiple data integration
BPNNHMDA (Li et al., 2020)	Based on a back propagation neural network design
GATMDA (Long et al., 2021)	Microbial Disease Association Prediction Using Graphical Attention Networks Combined with Matrix Filling
GCNMA (Wang et al., 2023)	Proposing a new computational model based on graph convolutional neural networks and multilayer attention mechanisms

The above methods can be categorised into four groups, with network-based methods constructing complex network structures based on known microbial disease associations. These network-based methods construct complex network structures based on known microbial-disease associations. Then, the potential probability of associations between microorganisms and diseases is inferred by analyzing the structural features of these networks and the lengths and numbers of connecting paths between the nodes (Wang et al., 2011). For example, Chen et al. proposed the KATZHMDA computational model to infer possible microbe-disease associations using the KATZ measure, which takes measurements to capture global information in a network by counting all paths between nodes and then predicts potential associations (Chen et al., 2017). However, the matrix decomposition-based approach focuses on decomposing the known microbe-disease association matrix into two feature matrices and approximating the original association matrix by the product of these two matrices (Ma and Liu, 2022). Information such as similarity and strength of association between microorganisms and diseases can be obtained. Shen et al. proposed a computational model of CMFHMDA based on synergistic matrix decomposition (Shen et al., 2017). In addition, based on the traditional machine learning approach by using known associations as training samples, we can construct a model for predicting the association between unknown microorganisms and diseases (Long et al., 2021). For example, Wang et al. designed the LRLSHMDA model which represents the network structure by constructing a Laplace matrix and predicts the association by regularized least squares (Wang et al., 2017). Finally, the graph neural network-based approach utilizes neural networks to take microbial and disease-related data as inputs and to extract and explore features and patterns from graph-structured data (Bessadok et al., 2022). They can utilize we can utilize the powerful learning ability of neural networks to discover potential associations between microbes and diseases and mine functional patterns and features from complex graph data. For example, Wang et al. designed GCNMA that captures structural information in the network by introducing a graph convolutional neural network and incorporates a multilayer attention mechanism to enhance the ability to model complex relationships between nodes (Wang et al., 2023).

Although the above models can perform reliably in some aspects, they still have limitations. For example, the computation of Katz path correlation must consider all paths between different nodes (Zhang et al., 2017). This may lead to high computational complexity on large datasets, especially when the network size is large (Kumar et al., 2020). In addition, regularized least squares usually introduce a regularization term to avoid overfitting. However, choosing the appropriate regularization parameter is not easy. If it is not chosen correctly, it may lead to underfitting or overfitting problems. When there is noise in the input data, the regularized least squares method may be too sensitive to the noise, resulting in unstable or inaccurate prediction results (Jung and Park, 2017).

### 3.4 Experimental results and analysis

In this section, we provide a detailed analysis of the experimental results of our proposed DuGEL model on the HMDAD and Disbiome datasets and compare it with nine other state-of-the-art microbe-disease association prediction methods. First, Tables 4, 5 show the performance of our proposed DuGEL model, and the nine compared methods on the HMDAD and Disbiome datasets, respectively, are mainly compared under the AUC assessment metrics. On the HMDAD dataset, the DuGEL model performs well, with its AUC values of 0.9698 and 0.9606 in 5-fold cross-validation and 2-fold cross-validation, respectively, The higher AUC values indicate the more vital overall predictive ability of the model, which indicates that DuGEL can effectively distinguish between positive and negative samples. For the Disbiome dataset, the DuGEL model still performs well. Under 5-fold cross-validation and 2-fold cross-validation settings, DuGEL reaches 0.9119 and 0.8932.

**TABLE 4:** HMDAD dataset in 5-fold cv and 2-fold cv.

Methods	AUC(5-fold cv)	AUC(2-fold cv)
KATZHMDA (Chen et al., 2017)	0.8291 ± 0.0041	0.8164 ± 0.0047
LRLSHMDA (Wang et al., 2017)	0.8792 ± 0.0032	0.8589 ± 0.0043
NTSHMDA (Luo and Long, 2018)	0.8893 ± 0.0043	0.8631 ± 0.0052
BiRWMP (Shen et al., 2018)	0.8777 ± 0.0089	0.8693 ± 0.0081
NBLPIHMDA (Wang et al., 2019)	0.8961 ± 0.0033	0.8792 ± 0.0056
HMDApred (Fan et al., 2020)	0.9357 ± 0.0041	0.9049 ± 0.0035
BPNNHMDA (Li et al., 2020)	0.9133 ± 0.0012	0.8949 ± 0.0023
GATMDA (Long et al., 2021)	0.9561 ± 0.0142	0.9541 ± 0.0053
GCNMA (Wang et al., 2023)	0.9610 ± 0.0223	0.9512 ± 0.0076
DuGEL (our model)	0.9698 ± 0.0172	0.9606 ± 0.0057

**TABLE 5:** Disbiome dataset in 5-fold cv and 2-fold cv.

Methods	AUC(5-fold cv)	AUC(2-fold cv)
KATZHMDA (Chen et al., 2017)	0.6781 ± 0.0132	0.6692 ± 0.0063
LRLSHMDA (Wang et al., 2017)	0.7361 ± 0.0221	0.7191 ± 0.0115
NTSHMDA (Luo and Long, 2018)	0.8301 ± 0.0059	0.8079 ± 0.0065
BiRWMP (Shen et al., 2018)	0.8319 ± 0.0092	0.8141 ± 0.0057
NBLPIHMDA (Wang et al., 2019)	0.8429 ± 0.0112	0.8281 ± 0.0142
HMDApred (Fan et al., 2020)	0.8521 ± 0.0381	0.8373 ± 0.0342
BPNNHMDA (Li et al., 2020)	0.8716 ± 0.0191	0.8532 ± 0.0151
GATMDA (Long et al., 2021)	0.9229 ± 0.0081	0.9201 ± 0.0141
GCNMA (Wang et al., 2023)	0.9001 ± 0.0161	0.8803 ± 0.0178
DuGEL (our model)	0.9119 ± 0.0059	0.8932 ± 0.0038

In comparison with previous methods, the DuGEL model demonstrates excellent performance. we attribute the results to the intrinsic structure of DuGEL. DuGEL successfully achieves efficient prediction of microbial-disease associations by combining a graph convolutional neural network (GCN) and a graph Attention Network (GAT), as well as introducing a Long Short-Term Memory Network (LSTM) to process fused features, enabling efficient prediction of microbe-disease associations. This multilevel feature extraction and sequence modeling approach enables DuGEL to perform well on key metrics (e.g., AUPR and AUC),demonstrating its robustness in microbe-disease association prediction tasks.

In particular, the DuGEL model combines the strengths of GCN and GAT; GCN can efficiently capture both local and global spatial features in the graph and extract complex relationships between microbes and diseases by performing convolutional operations on the features of neighboring nodes. Moreover, through the attention mechanism, GAT can assign different importance weights to its neighbors when processing nodes, thus better capturing the information of critical nodes. After combining the two, the outputs of GCN and GAT are fused through the Dual Graph Enhanced Layer of the DuGEL model, which effectively integrates the structural features and node importance.

Furthermore, introducing the LSTM module further enhances the model’s capabilities. LSTM is good at processing sequence data and can better identify potential associations between microbes and diseases by capturing temporal dependencies (Baranwal et al., 2022). The memory unit of LSTM can preserve information of long-time dependencies (Wu et al., 2021), which is especially important for analyzing potential microbe-disease relationships over long periods.

### 3.5 Ablation experiment

In addition, considering the role and contribution of every sub-module in the DuGEL model proposed in this study, we function ablation experiments on the HMDAD dataset to inspect the effect of distinctive parts of the model. We carried out three specific ablation experiments:

1. Doing away with the diagram convolution sublayer (denoted as “-GCN Layer”)
2. Removing the sketch attention sublayer (denoted as “-GAT Layer”)
3. Casting off the BiLSTM module (denoted as “-BiLSTM Module”)

Overall, each sub-module of the DuGEL model improves the primary mannequin’s effectiveness by taking pictures of the correlation between microorganisms and diseases. Table 6 suggests the results of the ablation experiments. In the first ablation experiment, after getting rid of the graph convolution sublayer, the mannequin’s AUC drops to 0.9465 and 0.9392 in 5-fold cross-validation and 2-fold pass validation, respectively, which suggests that GCN plays a crucial role in shooting nearby and international spatial points between microbes and diseases. By performing convolutional operations on the elements of neighboring nodes, GCN can extract complex correlation information, and removing this component considerably decreases the model’s predictive power.

**TABLE 6:** Fold cv and 2-fold cv and based on HMDAD dataset.

Methods	AUC(5-fold cv)	AUC(2-fold cv)
DuGEL (Proposed)	0.9698 ± 0.0172	0.9606 ± 0.0057
-GCN Layer	0.9465 ± 0.0185	0.9392 ± 0.0046
-GAT Layer	0.9520 ± 0.0163	0.9408 ± 0.0041
-BiLSTM Module	0.9602 ± 0.0053	0.9510 ± 0.0075

In the second ablation experiment, doing away with the GAT sublayer reduces the model’s AUC to 0.9520 and 0.9408 in 5-fold cross-validation and 2-fold pass-validation, respectively. GAT can better capture facts about key nodes via an attention mechanism that assigns unique weights of importance to its neighbors when processing nodes. This mechanism is necessary for improving the model’s prediction accuracy, and the mannequin’s performance in a similar fashion decreases after its removal.

Finally, we explored the function of the BiLSTM module. After eliminating the BiLSTM module, the AUC of the mannequin diminished to 0.9602 and 0.9510 in the 5-fold move validation and 2-fold move validation, respectively. The LSTM module appropriately processes sequence information and can become aware of attainable associations between microbes and diseases by capturing time dependencies. The reminiscence unit of the LSTM can retain lengthy time-dependent information, which is especially essential for examining the viable microbes’ disease. This is mainly necessary for analyzing doable microbe-disease relationships over lengthy periods. The elimination of this module resulted in a considerable reduction in the predictive energy of the model, in addition to demonstrating the necessary function of the BiLSTM module in the DuGEL model.

Overall, every sub-module had a practical impact on the expected performance of the DuGEL model. The GCN and GAT successfully extracted complicated associations between microbes and diseases through shooting spatial features and node importance statistics in the diagram structure, while the BiLSTM module furthermore desirable the predictive capacity of the mannequin via processing sequence features. The effects of the ablation experiments validate the effectiveness and necessity of these sub-modules and reveal the rationality and superiority of the DuGEL model sketch in the microbe-disease affiliation prediction task.3.6.

## 4 Case study

In this section, we selected three diseases, kidney stones, eczema, and ileal Crohn’s disease, as case studies for HMDAD to validate our model’s performance further. Specifically, we ranked these three relevant microorganisms in the prediction score and selected the top 20. Then, we evaluated the predictive performance of DuGEL by searching the literature. Among the common diseases, kidney stones cause severe back or abdominal pain accompanied by nausea, vomiting, hematuria, and other symptoms (Stevens, 2018). In recent years, kidney stones have been on the rise. It is prevalent in young adult males (Stamatelou and Goldfarb, 2023). A diverse microbial community exists around renal stones; changes in intestinal and urinary microorganisms may cause the occurrence and development of renal stones. *Clostridium difficile*, Bifidobacterium, and others are more closely associated with kidney stone occurrence (Miller et al., 2022). As shown in Table 7, the relevance of 17 of the top 20 candidate kidney stones-associated microorganisms predicted by DuGEL has been confirmed by previous publications.

**TABLE 7:** The top 20 Kidney stones microbes predicted by DuGEL.

Rank	Microbe	Evidence
1	*Clostridium difficile*	PMID: 30021693
2	*Helicobacter pylori*	PMID: 25459132
3	*Staphylococcus aureus*	PMID: 14241187
4	*Clostridium* coccoides	PMID: 37609403
5	*Staphylococcus*	PMID: 14241187
6	Bifidobacterium	PMID: 37145061
7	Comamonadaceae	Unconfirmeda
8	Oxalobacteraceae	PMID: 32381601
9	Sphingomonadaceae	PMID: 36970590
10	Dietzia maris	Unconfirmeda
11	*Staphylococcus* epidermidis	PMID: 20466,084
12	*Escherichia coli*	PMID: 14241187
13	*Acinetobacter*	PMID: 32111156
14	Corynebacterium	PMID: 24563271
15	Prevotella copri	PMID: 27708409
16	Propionibacterium	PMID: 33153435
17	Propionibacterium acnes	Unconfirmeda
18	Desulfovibrio	PMID: 38007438
19	Oxalobacter formigenes	PMID: 32880090
20	*Fusobacterium* nucleatum	PMID: 37458823

In addition, eczema is an inflammatory skin disease. According to many studies, microorganisms are strongly associated with eczema (Zimmermann et al., 2019). People with eczema have a less diverse and less stable skin microbiome than those without. This means the balance of beneficial and harmful bacteria on the skin is disrupted, making it more susceptible to infection and inflammation (Flowers and Grice, 2020). In addition, the presence of *Staphylococcus aureus* is associated with more severe eczema symptoms (Chapsa et al., 2023), suggesting a direct link between this bacteria and the condition. As shown in Table 8 below, it is evident that existing publications have confirmed 17 of the 20 potential eczema-associated microorganisms predicted by DuGEL.

**TABLE 8:** The top 20 Eczema microbes predicted by DuGEL.

Rank	Microbe	Evidence
1	*Clostridium difficile*	PMID: 27667310
2	*Helicobacter pylori*	PMID: 17568058
3	*Staphylococcus aureus*	PMID: 16965415
4	*Escherichia coli*	PMID: 27667310
5	Dietzia maris	PMID: 26821151
6	*Staphylococcus* epidermidis	PMID: 27416972
7	Stenotrophomonas maltophilia	PMID: 26821151
8	Comamonadaceae	Unconfirmeda
9	Oxalobacteraceae	PMID: 32971520
10	Sphingomonadaceae	PMID: 33735474
11	*Acinetobacter*	PMID: 28207943
12	Corynebacterium	PMID: 27562264
13	Prevotella copri	Unconfirmeda
14	Oxalobacter formigenes	Unconfirmeda
15	Desulfovibrio	PMID: 27812181
16	Tropheryma whipplei	PMID: 2,456,205
17	*Enterococcus*	PMID: 16601353
18	*Pseudomonas*	PMID: 33492004
19	*Staphylococcus*	PMID: 20222931
20	*Clostridium* coccoides	PMID: 24650346

Ileal Crohn’s disease is an inflammatory bowel disease. It causes swelling of the tissues of the digestive tract, which may lead to abdominal pain, severe diarrhea, fatigue, weight loss, and malnutrition (Fakhoury et al., 2014). The degree of symptoms ranges from mild to severe and usually comes on gradually, but sometimes, it can come on suddenly without warning. The cause of ileal Crohn’s disease is still unknown, but it is often assumed that a virus or bacteria may trigger Crohn’s disease. This paper presents a case study of ileal Crohn’s disease. As shown in Table 9, it is clear that 18 of the top 20 microorganisms associated with ileal Crohn’s disease have been confirmed in the published literature.

**TABLE 9:** The top 20 Ileal Crohn’s disease microbes predicted by DuGEL.

Rank	Microbe	Evidence
1	Actinobacteria	PMID: 33975420
2	Bacteroidetes	PMID: 32448900
3	*Clostridium* coccoides	PMID: 22719818
4	Firmicutes	PMID: 33975420
5	Prevotella	PMID: 35967326
6	Proteobacteria	PMID: 31530835
7	Enterobacteriaceae	PMID: 36268225
8	Lachnospiraceae	Unconfirmeda
9	*Bacteroides* ovatus	PMID: 26275394
10	*Bacteroides* uniformis	PMID: 33102745
11	*Bacteroides* vulgatus	PMID: 26275394
12	Clostridia	PMID: 30818349
13	Faecalibacterium prausnitzii	PMID: 18936492
14	*Clostridium* leptum	PMID: 16188921
15	*Lactobacillus*	PMID: 18839424
16	*Klebsiella*	PMID: 24223596
17	*Staphylococcus*	PMID: 23885156
18	Veillonella	Unconfirmeda
19	*Bacteroides*	PMID: 21484962
20	*Escherichia coli*	PMID: 15300573

## 5 Discussion and conclusion

Microorganisms play an important role in our lives and exist in countless numbers and diversity. Investigating the potential link between microorganisms and diseases cannot only contribute to the discovery of new therapeutic approaches and preventive strategies but also help advance the field of microbiology and medicine.

In this study, we propose a deep learning model called DuGEL to predict potential microbial disease associations. The DuGEL model combines graph convolutional neural network (GCN), graph attention network (GAT), and long-short-term memory network (LSTM) to efficiently capture and fuse the complex relationships between microbes and diseases. With the dual-channel structure, DuGEL can extract local and global features in the graph structure and enhance the model’s ability to capture critical nodes by assigning different importance weights to neighboring nodes through the attention mechanism. Our comprehensive experiments and case studies consistently show that DuGEL performs very satisfactorily in terms of prediction accuracy.

Although the DuGEL model has been very effective in studying the relationship between microbes and disease, it still has some limitations. Currently, the model relies heavily on the HMDAD and Disbiome datasets. Therefore, future work could focus on expanding the datasets to capture microbe-disease associations more comprehensively and accurately. In addition, the DuGEL model can be applied to drug-target interaction prediction and gene-disease association prediction to validate its broad applicability and effectiveness.

## Data Availability

The original contributions presented in the study are included in the article/supplementary material, further inquiries can be directed to the corresponding authors.
